# Genetic Variations of GAK in Two Chinese Parkinson’s Disease Populations: A Case-Control Study

**DOI:** 10.1371/journal.pone.0067506

**Published:** 2013-06-24

**Authors:** Wei-En Johnny Tseng, Chiung-Mei Chen, Yi-Chun Chen, Zhao Yi, Eng-King Tan, Yih-Ru Wu

**Affiliations:** 1 Department of Neurology, Chang Gung Memorial Hospital, Chang Gung University College of Medicine, Taipei, Taiwan; 2 Singapore General Hospital, National Neuroscience Institute, Singapore, Singapore; Kunming Institute of Zoology, Chinese Academy of Sciences, China

## Abstract

Cyclin G-associated kinase (GAK) modifies α–synuclein expression levels and affects the susceptibility of Parkinson’s disease (PD). The single-nucleotide polymorphism (SNP) rs1564282 of GAK gene has a significant association to the risk of PD among Caucasian populations. To date there is only one data with regards to ethnic Chinese from Mainland China. Here, we conducted a case-control study in two independent cohorts of Han Chinese populations from Taiwan and Singapore to validate this association. A total of 1,755 subjects (871 PD patients and 884 controls) were recruited. The results showed that neither the CT, TT genotypes nor the minor allele T of SNP rs1564282 were associated with PD among the subjects from Taiwan and Singapore as well as in the pooled analysis. Differences in our study population with regards to published literature may be due to epigenetic factors and gene-gene or gene-environmental interactions. Further studies in other Chinese populations will be of interest to validate these findings.

## Introduction

Parkinson’s disease (PD) is one of the most common neurodegenerative diseases, with a prevalence of ∼1% over 60 years of age [1]. It is characterized by the cardinal features of bradykinesia, muscular rigidity, 4–6 Hz resting tremor, postural instability and non-motor symptoms such as cognitive impairment, depression, anosmia, dysautonomia, sleep disorders [2], all of which can significantly decrease patient’s quality of life. Definite diagnosis of PD relies on pathology finding of α–synuclein immunopositive Lewy neurites and Lewy bodies [3]. However, PD is diagnosed typically based on clinical criteria, which is based on the presence of a combination of cardinal motor features, associated and exclusionary symptoms, and response to levodopa [2]. Damage to substantia nigra pars compacta (SNc), with severe obliteration of their neuromelanin-laden projection neurons, is frequently considered to be the most important hallmark of PD [3]. Moreover, mitochondrial dysfunction, oxidative stress, and protein mishandling play major roles in PD pathogenesis [1]. In search of disease etiology, gene mutations cause only a small proportion of all cases and that in most cases, non-genetic factors play a part, probably in interaction with susceptibility genes [1]. About 90% of cases are apparently sporadic, monogenetic mutations are now estimated to cause about 10% of PD cases [1]. Several pathogenic genes and susceptibility loci of Parkinson’s disease (PD) have been identified in both familial and sporadic cases in the last decade [4]. Genome-wide association studies (GWAS) have identified several susceptible genes contributing to PD. Thirteen loci showed genome-wide significant (p<5×10^−8^) association with disease risk: BST1, CCDC62/HIP1R, DGKQ/GAK, GBA, ITGA8, LRRK2, MAPT, MCCC1/LAMP3, PARK16, SNCA, STK39, SYT11/RAB25, and RIT2 have been found [5]–[7].

Cyclin G-associated kinase (GAK) is a 160 kDa serine/threonine kinase, located p16.3 on chromosome 4. It is a regulator of clathrin-mediated membrane traffic, also controls centrosome integrity and chromosome congression [8]. Its’ vital role in cell proliferation and receptor trafficking was revealed in osteosarcoma [9]. It has been shown to up regulate genes in SNc of Parkinson’s disease [10]. The clathrin-binding C-terminal domain of GAK binds to pre-cathepsin D (CTSD), which is implicated as the main lysosomal enzyme involved in α–synuclein degradation [11]–[13]. CTSD mutations can induce the pathological accumulation of α–synuclein. Taken together, GAK modifies α–synuclein expression levels and toxicity in PD, that reduced GAK function enhances α–synuclein-mediated toxicity [14]. Recently, a significant association between the single-nucleotide polymorphism (SNP) rs1564282 of GAK gene and risk of PD was described in several Caucasian studies [15]–[19], and only one in Han Chinese from Mainland China [20]. To understand the genetic effects across different populations, we aimed to investigate the genetic susceptibilities of SNP rs1564282 in GAK among Han Chinese in the Taiwan and Singapore populations.

## Methods

### Patient population and Ethics statement

Patients diagnosed with PD were recruited from the neurology clinics of the Chang Gung Memorial Hospital (CGMH) in Taiwan and Singapore General Hospital in Singapore. The diagnosis of PD was based on the UK PD Society Brain Bank clinical diagnostic criteria [21]. Healthy adult volunteers, as the control group, were matched for age, gender, ethnic origin, and area of residences. This study was performed according to a protocol approved by the institutional review boards of Chang Gung Memorial Hospital (ethical license No:101-2097C), and all examinations were performed after obtaining written informed consents. This study also approved by Singhealth Institution Review Board in Singapore. Written informed consent was obtained from every subject too.

### Genetic analysis

In Taiwan’s cohort, genotyping of the GAK SNP (rs1564282) was performed using TaqMan® Assays commercially available (Applied Biosystems Inc., California, USA). TaqMan® PCR was performed according to the manufacturer’s standard PCR protocol. Briefly, 20 ng genomic DNA was mixed with the supplied 2X TaqMan Universal PCR Master Mix No AmpErase UNG and 40X TaqMan Assay Mix to a final volume of 5μl in a 384-well plate. Each sample underwent 50 amplification cycles on GeneAmp® PCR System 9700 (Applied Biosystems Inc.). Fluorescent signals of the two probes were analyzed end-point fluorescent data on ABI PRISM® 7900HT Sequence Detection System (Applied Biosystems Inc.). Genotype was determined automatically by Sequence Detection Software (Applied Biosystems Inc.).

In Singapore’s cohort, this SNP was screened on a MALDI-TOF mass spectrometry using the Sequenom MassARRAYTM system (San Diego, CA). Briefly, multiplex genotyping assays were designed using the Sequenom DESIGNER software (San Diego, CA). Initial PCR (5 ng of genomic DNA) and primer extension reactions were carried out according to the Sequenom genotyping assay iPLEX^TM^ protocol. After purification, 15 nl of primer extension product was analyzed with a MassARRAY Sequenom-Bruker Spectrometer (Bruker Biosciences, San Diego, CA). Sequence analysis was used to confirm the genotypes for representative samples. Dideoxy chain terminator sequencing was carried out according to the manufacturers’ instructions (BigDye, Applied Biosystems, Inc.) and the products were electrophoresed on an ABI 3100 automated DNA sequencer (Applied Biosystems, Inc.). Although the genotyping methods were different between Taiwan and Singapore cohorts, the consistency of the methods was verified by sequencing.

### Statistical analysis

Chi square test was used to compare the frequency of the allele and genotypes in both cases and controls. The genotypes of the cases and controls followed the Hardy Weinberg equilibrium. Odds ratios and 95% confidence intervals were estimated [22]. Power calculation was carried out using QUANTO software [23], ver. 1.2.4. In the present case-control study, at the 5% significance level, we had a power greater than 0.8 to identify an association when the per-allele genetic effect was greater than an odds ratio of 1.3 and the disease allele frequency was greater than 0.1.

## Results

A total of 1,755 subjects (871 PD patients and 884 controls) from Taiwan and Singapore were included. From Taiwan, the study enrolled 483 patients (46.2% females) diagnosed with PD by two neurologists that specialized in movement disorders (YR Wu and CM Chen) in Taiwan. Only one proband with familial PD cases in the same family was recruited. The mean age at onset (AAO) of PD symptoms was 63.0±11.2 years (range 19∼93) and the mean age of recruitment of 495 controls (53.1% female) was 60.2±13.2 years (range 17∼91). The frequencies of rs1564282 genotypes and alleles were similar in both PD patients and controls (Table 1).

**Table 1:** Frequency of Genotype and Allele Polymorphisms of GAK rs1564282 among PD and Controls in Taiwan, Singapore and China.

	PD (%)	Controls (%)	OR (95% CI)	*P*-value
Taiwan				
CC	381(78.9)	387(78.2)	1.00	
CT	97(20.1)	104(21.0)	0.95(0.69–1.29)	0.73
TT	5(1.0)	4(0.8)	1.27(0.32–5.35)	0.74
CT+TT	102(21.1)	108(21.8)	0.96(0.71–1.30)	0.79
Common (C) allele	859(88.9)	878(88.7)	1.00	
Minor (T) allele	107(11.1)	112(11.3)	0.98(0.74–1.29)	0.87
Singapore				
CC	306(78.9)	311(79.9)	1.00	
CT	77(19.8)	77(19.8)	1.02(0.71–1.45)	0.93
TT	5(1.3)	1(0.3)	5.07(0.69–121.3)	0.12
CT+TT	82(21.1)	78(20.1)	1.06(0.75–1.51)	0.71
Common (C) allele	689(88.8)	699(89.8)	1.00	
Minor (T) allele	87(11.2)	79(10.2)	1.12(0.81–1.54)	0.50
Merged (Taiwan + Singapore)				
CC	687(78.9)	698(78.9)	1.00	
CT	174(20.0)	181(20.5)	0.98(0.77–1.23)	0.84
TT	10(1.1)	5(0.6)	2.03(0.69–6.61)	0.20
CT+TT	184(21.1)	186(21.1)	1.01(0.80–1.27)	0.99
Common (C) allele	1548(88.9)	1577(89.2)	1.00	
Minor (T) allele	194(11.1)	191(10.8)	1.035(0.84–1.28)	0.75
China				
CC	616(75.9)	616(80.8)	1.00	
CT	183(22.5)	142(18.6)	1.29(1.01–1.65)	0.04
TT	13(1.6)	4(0.5)	3.25(1.10–11.61)	0.03
CT+TT	196(24.1)	146(19.2)	1.3(1.05–1.71)	0.02
Common (C) allele	1415(87.1)	1374(90.2)	1.00	
Minor (T) allele	209(12.9)	150(9.8)	1.36(1.09–1.69)	0.007
Merged (Taiwan + Singapore + China)				
CC	1303(77.4)	1314(79.8)	1.00	
CT	357(21.2)	323(19.6)	1.12(0.94–1.32)	0.21
TT	23(1.4)	9(0.6)	2.58(1.21–5.88)	0.01
CT+TT	380(22.6)	332(20.0)	1.15 (0.9–1.36)	0.09
Common (C) allele	2963(88.0)	2951(89.6)	1.00	
Minor (T) allele	403(12.0)	341(10.4)	1.18(1.01–1.37)	0.04

P-values and OR calculated in relation to CC genotype and a major allele C.

All p-values were calculated by means of chi-squared test.

In the Singapore cohort, there were 388 PD and 389 controls. The mean AAO of the 388 PD patients (44% female) was 63.0±10.0 years (range 21–90) and age at sampling of 389 controls (47.0% female) was 60.0±10.5 years (range 20–90). Data for the GAK SNP regarding the eighty cases and fifty controls had been previously used in another study [24].

There were also no significant differences between genotypes and alleles in the Singapore’s cohort, even though there was a slight increase in the odds ratio of 1.12. A pooled analysis of our two Asian cohorts also did not reveal any significant differences between PD and controls (Table 1). Stratification by age at onset (<50 and ≧ 50 years) and sex also did not show differences in the minor T allele frequency in each case control cohort (data not shown). We had a power greater than 0.8 to identify an association at 5% significance level for our case-control study.

## Discussion

GAK was first recognized as the susceptible gene associated with PD in a cohort of familial PD study [15]. Subsequent studies showed the association of GAK in PD risk appears to be present in sporadic PD as well [16]–[19]. However, all the studies were done utilizing primarily Caucasian populations such as Americans, the British, the French, and the Dutch [15]–[19]. Across different populations, our study showed the SNP rs1564282 was not associated with PD among Han Chinese in Taiwan and Singapore. There was no significant difference neither in CT, TT genotypes nor minor allele T. Pooled analysis of our two Asian cohorts did not reveal any significant differences between PD and controls.

In contrast, one study conducted in Mainland China showed that the rs1564282 variant of GAK increases the risk of PD in Han Chinese. The minor allele displayed an OR = 1.36 (95% CI = 1.09, 1.69, P = 0.007), and CT + TT genotypes had an OR of 1.34 (95% CI = 1.05, 1.72, P = 0.017) in their cohort [20]; our combined data showed the minor allele had an OR = 1.035 (95% CI = 0.84, 1.28, P = 0.75), and CT + TT genotypes displayed an OR of 1.005 (95% CI = 0.80, 1.27, P = 0.99). The genotyping characteristic was different in these two study populations. The minor allele has higher frequency in cases (12.9% vs 11.1%) and lower in controls (9.8% vs 10.8%) than our merged data. The subjects enrolled in this study were much younger than our study, in which the mean age of PD patients and controls were 53.60±11.38 and 53.77±14.37, respectively. After pooling all three sets of data, only the allele frequency showed a marginal significance, OR =  1.18 (95% CI = 1.01, 1.37, P = 0.04), and CT + TT genotypes did not reach a level of significant difference, OR = 1.15 (95% CI = 0.9–1.36, P = 0.09) (Table 1). Further studies in other Han Chinese populations would be of interest.

The roles of epigenetic factors, gene-gene or gene-environmental interactions have not been evaluated and they could be confounding variables. One potential issue is that our power calculation was based on the assumption that the typed SNP is the causal SNP. At this moment, we do not fully understand the linkage disequilibrium structure of the unknown casual SNP and hence further replication studies may be required to determine if the actual casual SNP is identified. Moreover, only one SNP rs1564282 was analyzed which does not rule out the association of other regions within or around the gene, such as rs11248051, rs6599388, chr4:911311. In addition, such differences may be due to population-specific heterogeneity in PD [24].

In conclusion, we showed that GAK SNP rs1564282 does not contribute to the risk of PD among Han Chinese in Taiwan and Singapore. However, further studies of this and other SNPs within the GAK gene in other ethnic Chinese populations may be useful to validate our findings.
